# Supplementary material for: Stoichiometry-engineered phase transition in a two-dimensional binary compound
Source: Nat Commun. 2025 May 5;16:4162. doi: 10.1038/s41467-025-59429-3 (PMC12052965; doi:10.1038/s41467-025-59429-3)
Supplement: Supplementary file 1 — Supplementary Information [file 41467_2025_59429_MOESM1_ESM.pdf]

Supplementary Information for

Stoichiometry-engineered phase transition in a two-dimensional binary compound

Mengting Huang[1#], Ze Hua[2#], Roger Guzman[3#], Zhihui Ren[4], Pingfan Gu[5,6], Shiqi Yang[5], Hui Chen[1], Decheng Zhang[1], Yiming Ding[1], Yu Ye[5], Caizhen Li[4,7], Yuan Huang[1,8*], Ruiwen Shao[2*], Wu Zhou[3*], Xiaolong Xu[1*], Yeliang Wang[1*]

Corresponding Authors: yhuang@bit.edu.cn; rwshao@bit.edu.cn; wuzhou@ucas.ac.cn; xuxiaolong@bit.edu.cn; yeliang.wang@bit.edu.cn

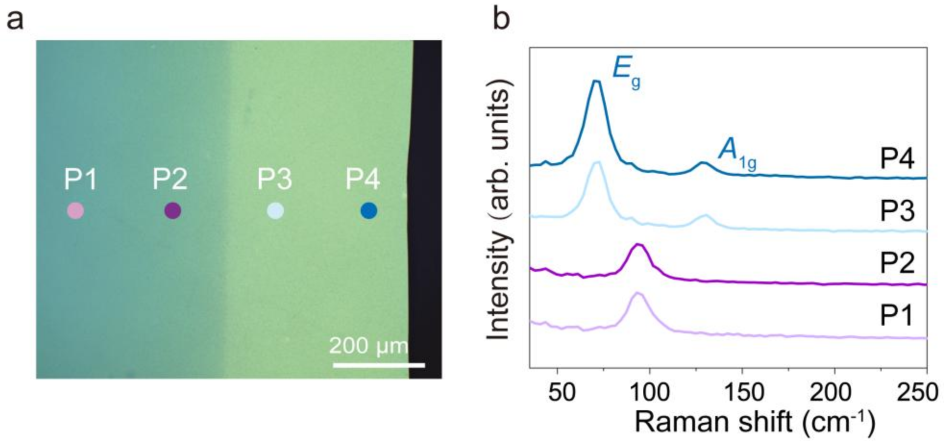

Supplementary Fig. 1 Characterizations of the PdTe and $PdTe_2$ film synthesized from 5 nm Pd film by in-situ CVD system. a, In contrast to the tellurization results of 10 nm and 20 nm Pd thin films, no obvious nucleation process is observed, and only these two kinds of contrasts are found. **b**, Typical Raman spectra collected at different positions, marked by the points at Supplementary Fig. 1a. At the edge of silicon (P3 and P4), the collected Raman spectra showed two peaks at ~75 cm^{-1} and 133 cm^{-1}, associated with the in-plane E_g and the out-of-plane A_{1g} vibration modes of $PdTe_2$. Another contrast away from the edge of the silicon (P1 and P2) showed new Raman peak arose at ~98cm^{-1} which is the characteristic Raman peak of PdTe crystals.

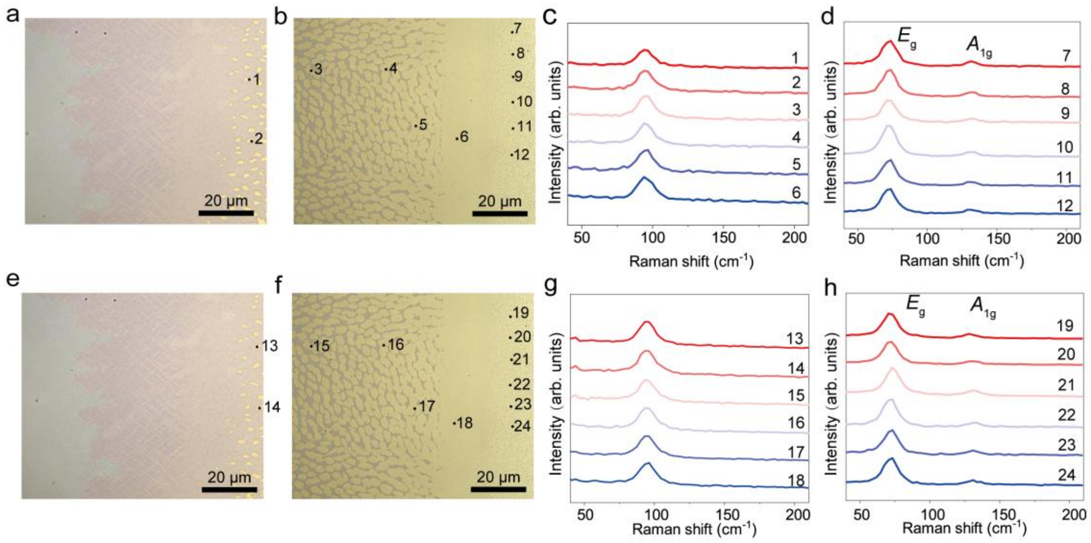

Supplementary Fig. 2 The thermodynamic stability of each phase. a-d, The optical micrograph of the sample before annealing and the corresponding Raman spectra. **e-h**, The optical micrograph of the sample after annealing at 500°C and the corresponding Raman spectra.

a

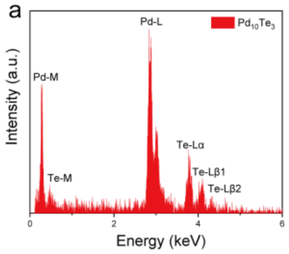

Z	Element	Atomic fraction (%)	Atomic error (%)	Mass fraction (%)	Mass error (%)	Fit error
46	Pd	76.54	7.71	75.33	5.35	0.59
52	Te	23.46	2.99	24.67	3.01	1.40

b

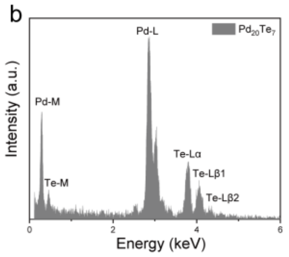

Z	Element	Atomic fraction (%)	Atomic error (%)	Mass fraction (%)	Mass error (%)	Fit error
46	Pd	73.50	7.53	73.08	5.20	0.43
52	Te	26.50	3.27	26.92	3.27	0.55

c

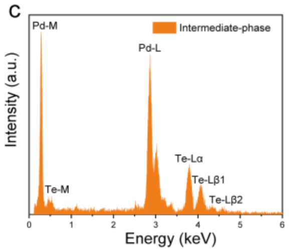

Z	Element	Atomic fraction (%)	Atomic error (%)	Mass fraction (%)	Mass error (%)	Fit error
46	Pd	68.66	7.30	68.91	4.98	0.52
52	Te	31.34	3.85	31.09	3.80	0.82

d

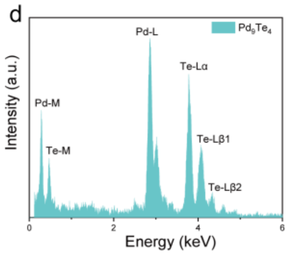

Z	Element	Atomic fraction (%)	Atomic error (%)	Mass fraction (%)	Mass error (%)	Fit error
46	Pd	68.24	7.28	68.46	4.96	0.63
52	Te	31.76	3.91	31.54	3.86	0.97

e

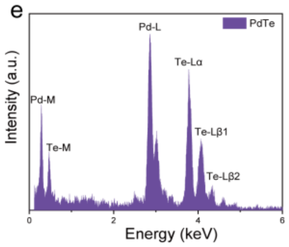

Z	Element	Atomic fraction (%)	Atomic error (%)	Mass fraction (%)	Mass error (%)	Fit error
46	Pd	50.98	6.39	50.46	4.05	0.40
52	Te	49.02	6.86	49.54	6.29	0.66

f

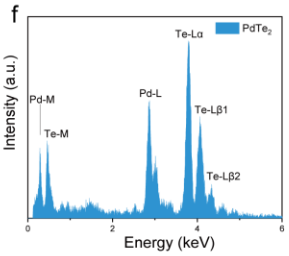

Z	Element	Atomic fraction (%)	Atomic error (%)	Mass fraction (%)	Mass error (%)	Fit error
46	Pd	33.33	5.23	33.19	3.07	0.38
52	Te	66.67	10.72	66.81	9.01	0.48

Supplementary Fig.3 EDS spectrum, elemental composition and ratio of a, $Pd_{10}Te_3$, **b**, $Pd_{20}Te_7$, **c**, intermediate phase, **d**, Pd_9Te_4, **e**, PdTe and **f**, $PdTe_2$ phases.

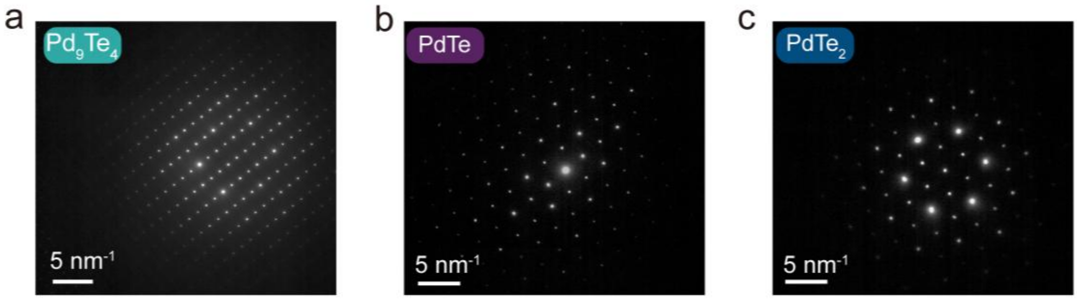

Supplementary Fig. 4 SAED characterizations of **a**, Pd_9Te_4, **b**, PdTe and **c**, $PdTe_2$ phases.

Supplementary Table 1 Crystallographic data of $Pd_{10}Te_3$ crystals from SC-XRD experiments.[1-2]

Crystal system		Cubic		
Space group		$Fd\bar{3}m$ (No. 227)		
Lattice paramenters (Å)		a = b = c=12.65, α= β= γ = 90°		
Cell volume (Å^3)		2023.93		
Density (g cm^{-3})		9.50		
Structure paramenters:				
Element	Wyckoff	x	y	z
Te	8b	1/4	3/4	1/4
Te	16c	3/8	1/8	3/8
Pd	32e	0.115882	0.384118	0.115882
Pd	48f	1/4	1/4	0.00809

The data used in this study were obtained from the Material Project database (https://materialsproject.org), and the Material ID is [mp-30067].

Supplementary Table 2 Crystallographic data of Pd_9Te_4 crystals from SC-XRD experiments.[1, 3-5]

Crystal system		Monoclinic		
Space group		$P2_1/c$ (No. 14)		
Lattice paramenters (Å)		a=7.52, b=14.09, c=8.96, α=90°, β=91.6°, γ = 90°		
Cell volume (Å^3)		949.60		
Density (g cm-3)		10.27		
Structure paramenters:				
Element	Wyckoff	x	y	z
Te	4e	0.246524	0.995829	0.147101
Te	4e	0.294897	0.494888	0.113104
Te	4e	0.047435	0.737378	0.357536
Te	4e	0.461518	0.248108	0.433593
Pd	4e	0.206546	0.677802	0.104741
Pd	4e	0.275849	0.3065	0.175711
Pd	4e	0.041198	0.938161	0.387449
Pd	4e	0.110007	0.547533	0.358382
Pd	4e	0.011344	0.349261	0.391266
Pd	4e	0.168563	0.141472	0.362191
Pd	4e	0.345317	0.828683	0.298418
Pd	4e	0.501477	0.631498	0.306679
Pd	4e	0.415786	0.43527	0.394243

The data used in this study were obtained from the Material Project database (https://materialsproject.org), and the Material ID is [mp-29907].

Supplementary Table 3 Crystallographic data of PdTe crystals from SC-XRD experiments.[1, 3, 6-9]

Crystal system		Hexagonal		
Space group		*P*6_3/*mmc* (No. 194)		
Lattice paramenters (Å)		a = b =4.18, c=5.74, α= β=90°, γ = 120°		
Cell volume (Å^3)		86.91		
Density (g cm^{-3})		8.94		
Structure paramenters:				
Element	Wyckoff	x	y	z
Te	2a	0	0	0
Pd	2c	1/3	2/3	1/4

The data used in this study were obtained from the Material Project database (https://materialsproject.org), and the Material ID is [mp-564].

Supplementary Table 4 Crystallographic data of $PdTe_2$ crystals from SC-XRD experiments. [1, 6,7,9-13]

<table>
<tr><td colspan="2">Crystal system</td><td colspan="3">Trigonal</td></tr>
<tr><td colspan="2">Space group</td><td colspan="3">$P\bar{3}m1$ (No. 164)</td></tr>
<tr><td colspan="2">Lattice paramenters (Å)</td><td colspan="3">a = b = 4.07, c=5.23，
α= β=90°, γ = 120°</td></tr>
<tr><td colspan="2">Cell volume ($Å^3$)</td><td colspan="3">75.08</td></tr>
<tr><td colspan="2">Density (g cm^{-3})</td><td colspan="3">8.00</td></tr>
<tr><td colspan="5">Structure paramenters:</td></tr>
<tr><td>Element</td><td>Wyckoff</td><td>x</td><td>y</td><td>z</td></tr>
<tr><td>Te</td><td>1a</td><td>0</td><td>0</td><td>0</td></tr>
<tr><td>Pd</td><td>2d</td><td>1/3</td><td>2/3</td><td>0.261091</td></tr>
</table>

The data used in this study were obtained from the Material Project database (https://materialsproject.org), and the Material ID is [mp-782].

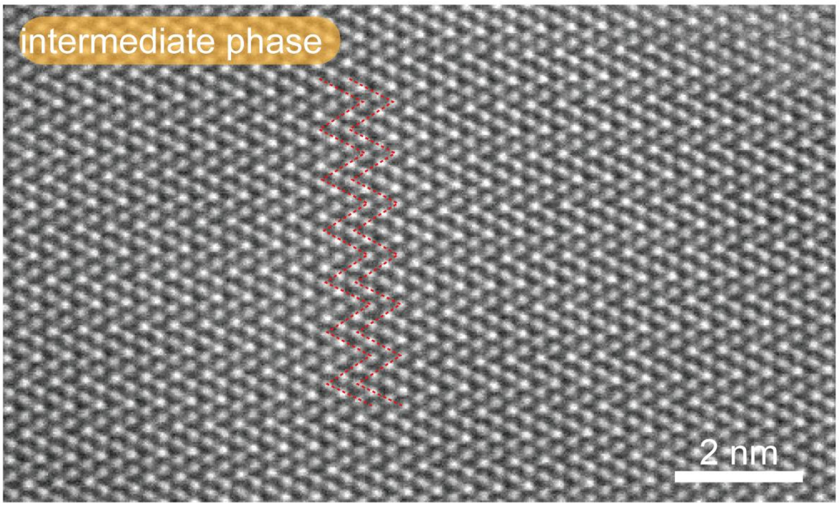

Supplementary Fig. 5 Atomic-resolution HAADF-STEM characterization of intermediate phase.

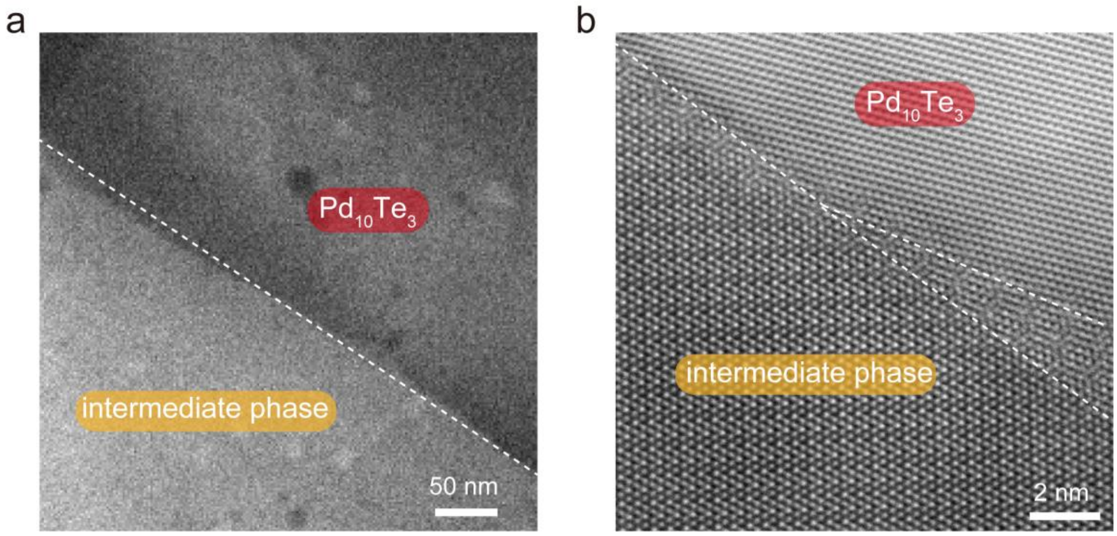

Supplementary Fig. 6 STEM characterization of phase boundary between $Pd_{10}Te_3$ and intermediate phase. a, Low-magnification STEM image of the phase boundary. **b**, Atomic-resolution HAADF-STEM image of the interface between $Pd_{10}Te_3$ and intermediate phase.

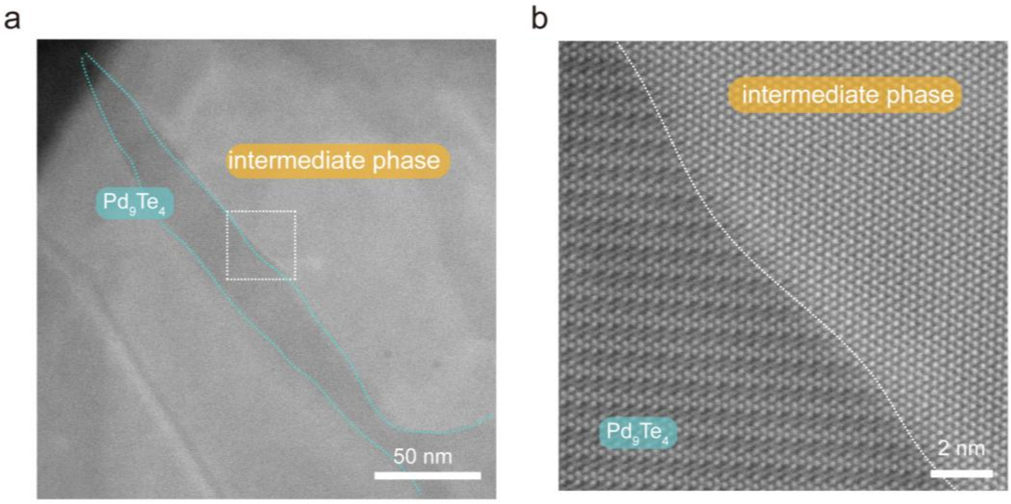

Supplementary Fig. 7 STEM characterization of phase boundary between intermediate phase and Pd_9Te_4. a, Low-magnification STEM image of the phase boundary. The Pd_9Te_4 phase appear in strip shape. **b**, Atomic-resolution HAADF-STEM image of the interface between Pd_9Te_4 and intermediate phase.

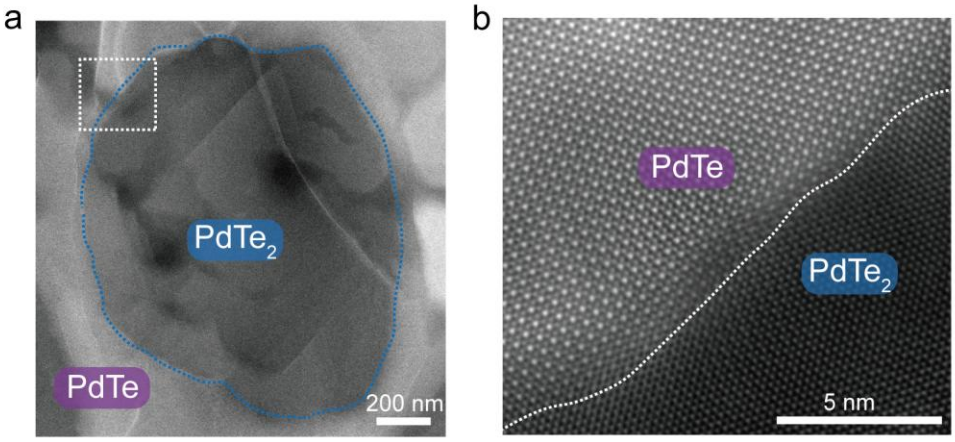

Supplementary Fig. 8 STEM characterization of phase boundary between PdTe and $PdTe_2$. a, Low-magnification STEM image of the phase boundary. The $PdTe_2$ phase appear in circular shape. **b**, Atomic-resolution HAADF-STEM image of the interface between PdTe and $PdTe_2$.

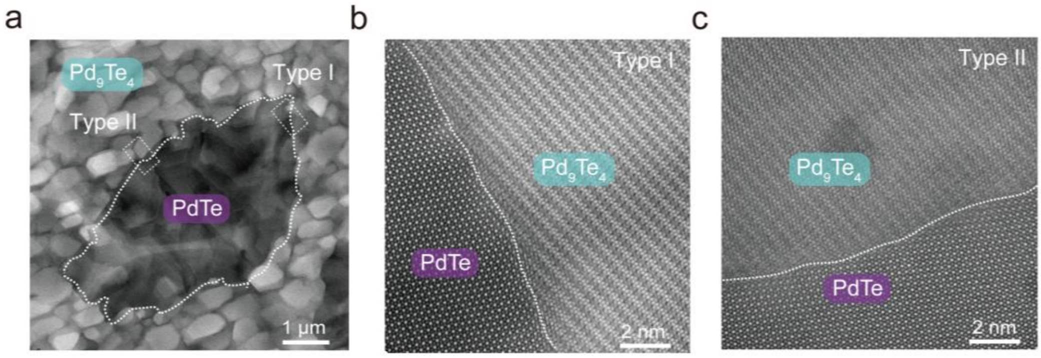

Supplementary Fig. 9 STEM characterization of phase boundary between Pd_9Te_4 and PdTe. a, Low-magnification STEM image of the phase boundary. The PdTe phase appears in elliptical shapes, the interface in two directions have been characterized respectively, named type I and type II. **b**, Atomic-resolution HAADF-STEM image of the type-I interface. **c**, Atomic-resolution HAADF-STEM image of the type-II interface.

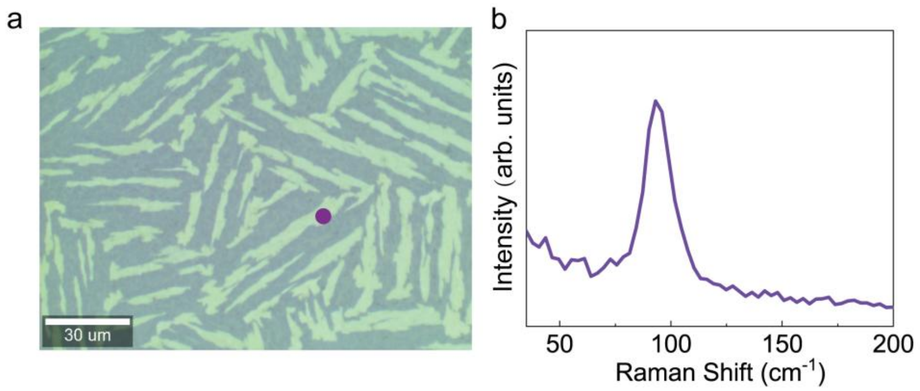

Supplementary Fig.10 Optical image (a) and Raman spectrum (b) of the elongated PdTe nucleations.

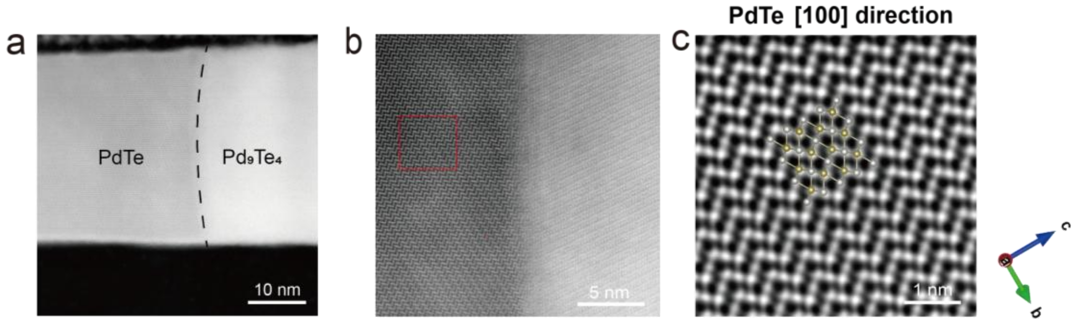

Supplementary Fig. 11 Cross-sectional STEM characterization of the interface between the elongated PdTe nucleation and Pd_9Te_4 background. a, The cross-sectional STEM image at low magnification shows that the two phases have different contrasts and are seamlessly connected together. **b**, The atomic-resolved cross-sectional STEM image. **c**, The enlarged atomic-resolved HAADF-STEM image of PdTe phase, showing a tilted c axis.

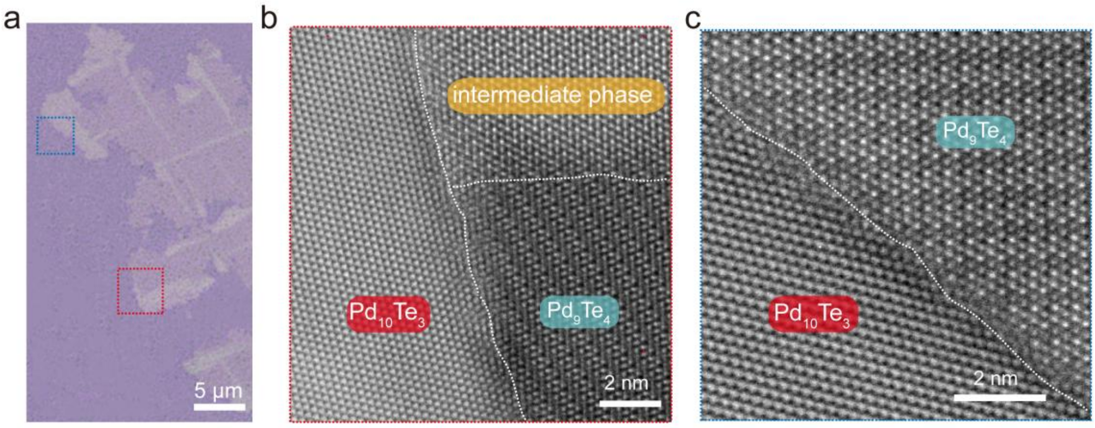

Supplementary Fig. 12 STEM characterization of phase boundary between $Pd_{10}Te_3$, intermediate phase and Pd_9Te_4. a, Optical image of the synthesized film. There is the interface of $Pd_{10}Te_3$, intermediate phase and Pd_9Te_4 in the red box. There is the interface of $Pd_{10}Te_3$ and Pd_9Te_4 in the bule box. **b**, Atomic-resolution HAADF-STEM image of the interface of $Pd_{10}Te_3$, intermediate phase and Pd_9Te_4. **c**, Atomic-resolution HAADF-STEM image of the interface of $Pd_{10}Te_3$ and Pd_9Te_4.

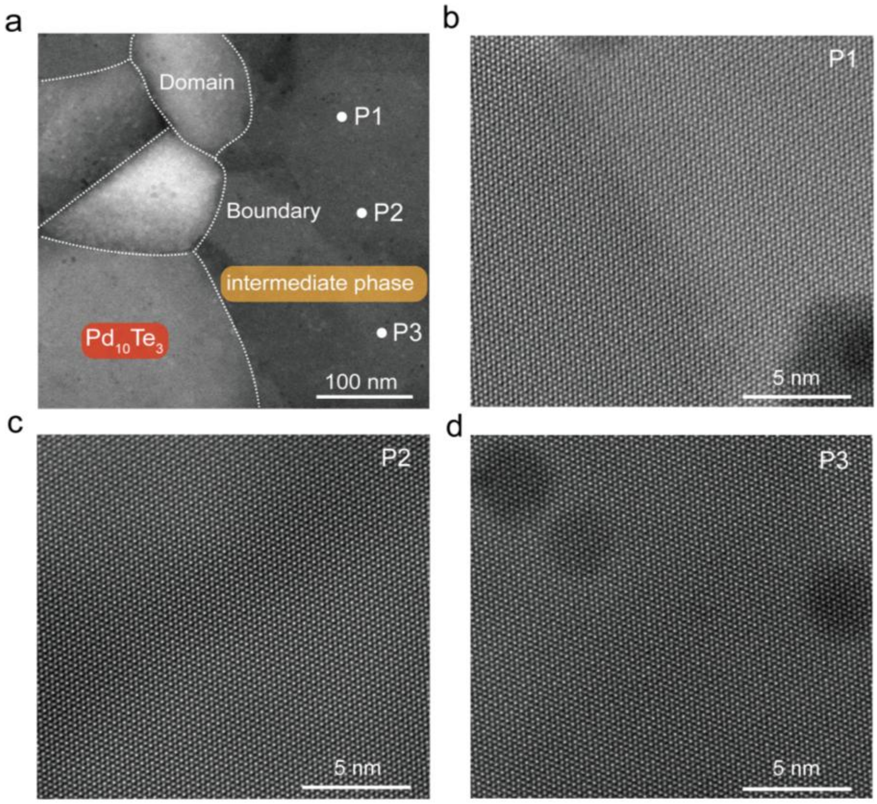

Supplementary Fig. 13 STEM characterization of intermediate phase in one domain. a, Low-magnification STEM image of the phase boundary of $Pd_{10}Te_3$ and intermediate phase. **b-d**, Atomic-resolution HAADF-STEM images of intermediate phase at different positions marked by the white points in Supplementary Fig. 13a.

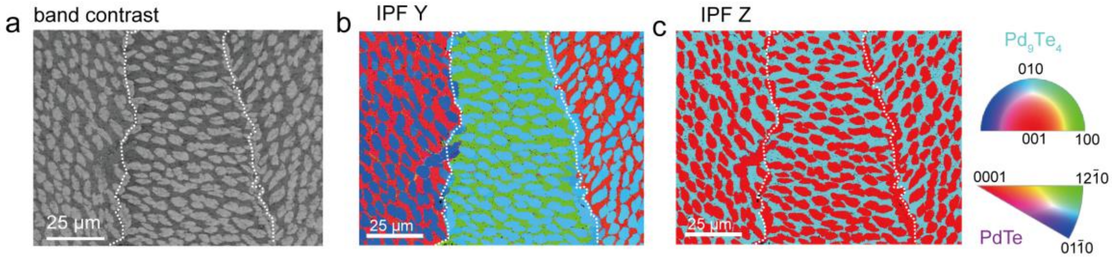

Supplementary Fig. 14 EBSD characterization of the interface of Pd_9Te_4 and PdTe in three different Pd_9Te_4 domains. a, Band contrast image of the PdTe nucleations region, and the contrast originates from the local intensity variations of Kikuchi patterns. The PdTe nucleations exhibit brighter contrast, indicating good crystallinity. Based on the orientation of the PdTe nucleations, this region can be divided into three parts labeled by the white dashed lines. **b**, IPF Y map of the interface. The IPF map around three different Pd_9Te_4 domains, showing three distinct contrasts of Pd_9Te_4 phases and the circle nucleation (PdTe) along the interface. This is because the crystal orientation difference of Pd_9Te_4 causes that of PdTe in the process of phase transformation. The outline of the three different Pd_9Te_4 domains is marked by the white dashed line. **c**, IPF Z map of the interface. The uniform cyan color of Pd_9Te_4, related to the [010] crystal orientation, indicates that Pd_9Te_4 thin film stacks along the direction normal to the surface. The uniform red color of PdTe, related to the [0001] crystal orientation, indicates that PdTe thin film stacks along the direction normal to the surface.

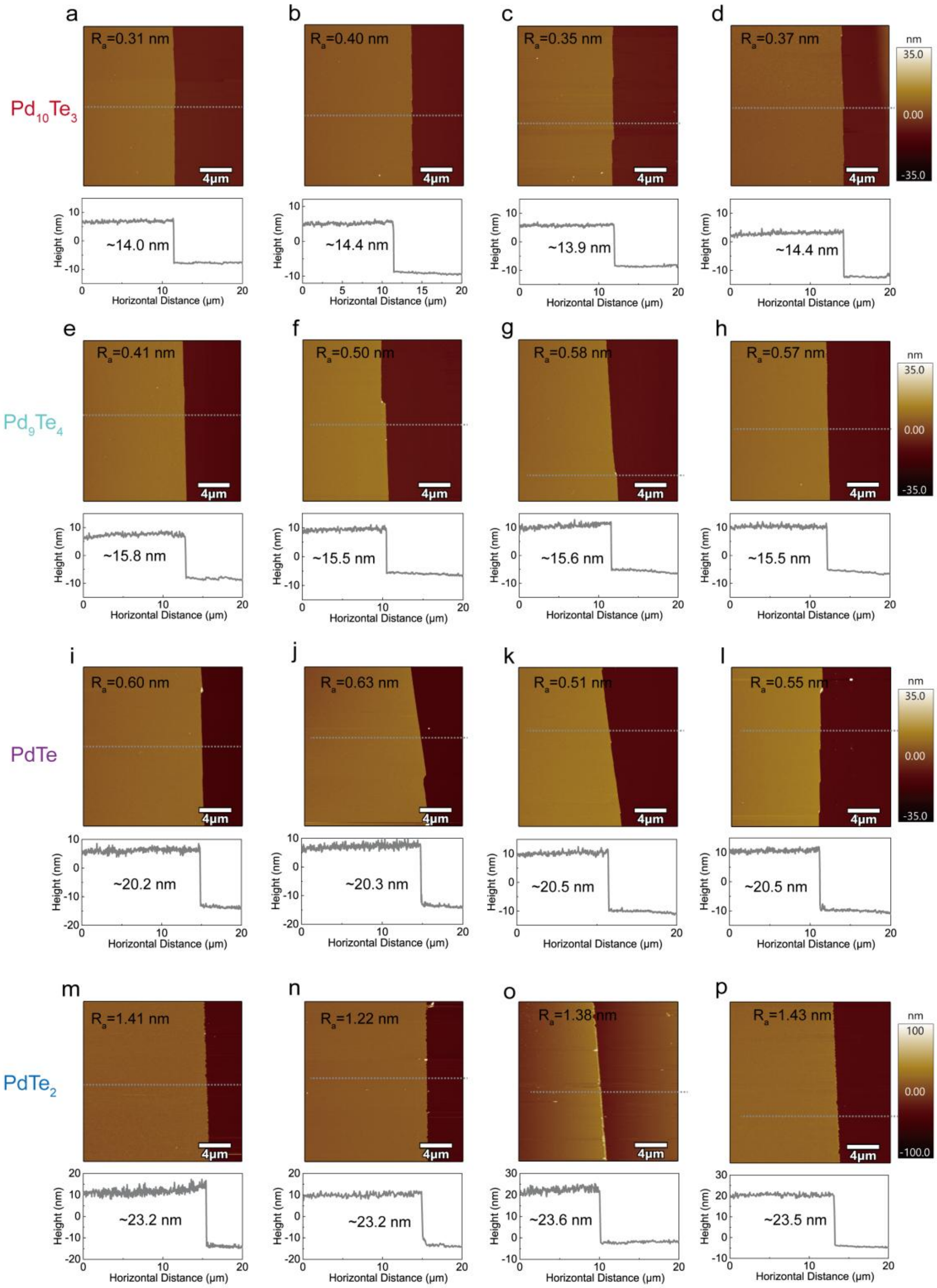

Supplementary Fig. 15 AFM height images and corresponding curve of $Pd_{10}Te_3$, Pd_9Te_4, PdTe and $PdTe_2$ wafer measured at different positions respectively, indicating that the thickness of the film has good uniformity over a large scale. a-d, $Pd_{10}Te_3$, **e-h**, Pd_9Te_4, **i-l**, PdTe, **m-p**, $PdTe_2$.

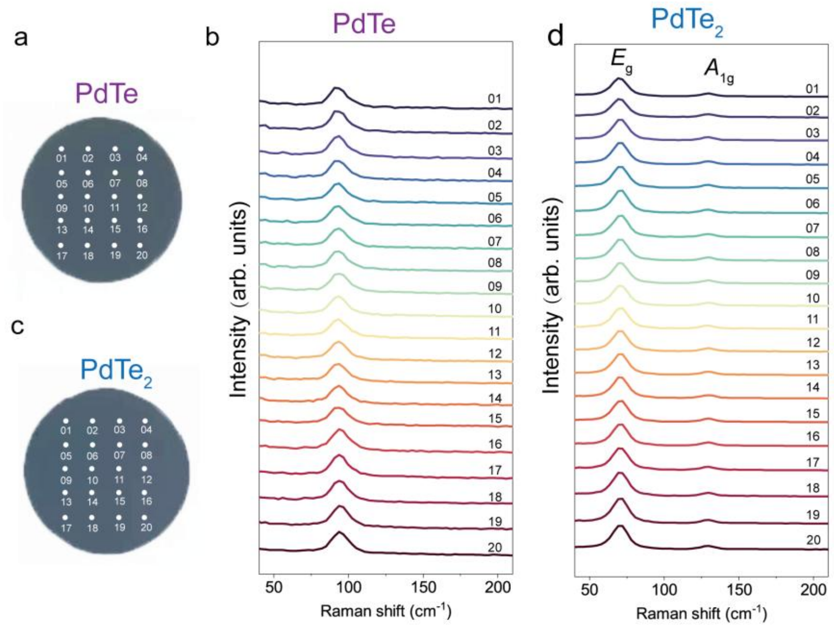

Supplementary Fig. 16 The Raman spectra of PdTe and $PdTe_2$ wafer. a-b, The optical micrograph of PdTe wafer and the corresponding Raman spectra. **c-d**, The optical micrograph of $PdTe_2$ wafer and the corresponding Raman spectra. The Raman characteristic peaks indicate the film has good uniformity over a large scale.

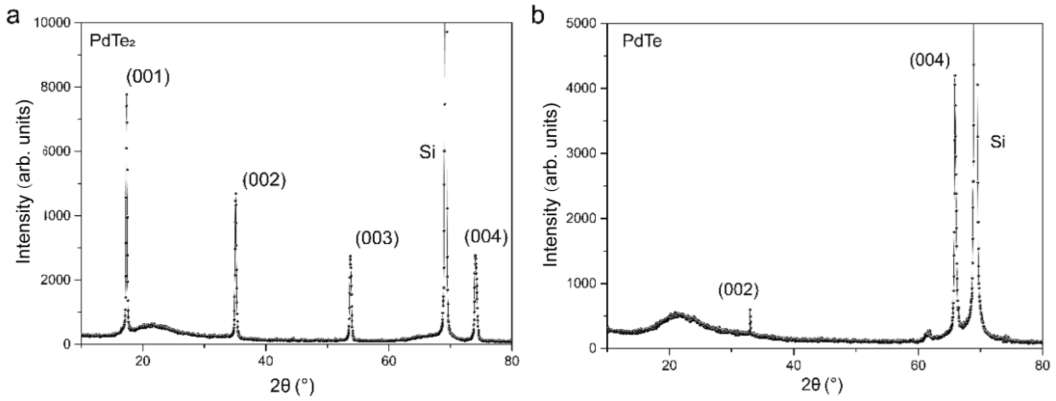

Supplementary Fig. 17 The XRD spectra of **a**, $PdTe_2$ and **b**, PdTe.

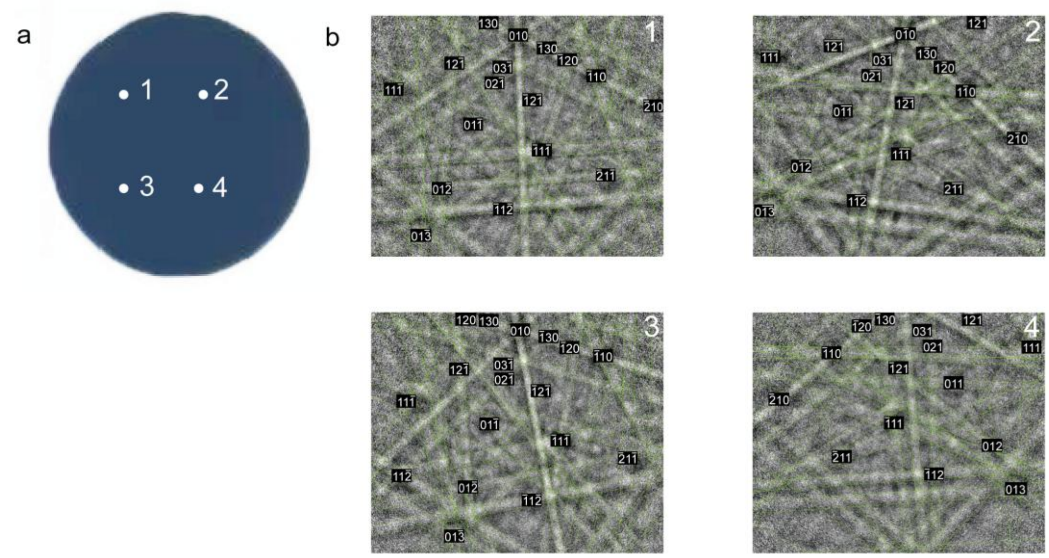

Supplementary Fig. 18 Kikuchi pattern diffractions of the Pd_9Te_4 wafer using EBSD. a, The optical images of Pd_9Te_4 wafer. **b**, Clear Kikuchi pattern diffractions could be seen at different positions, indicating that the films at different positions had good crystallinity.

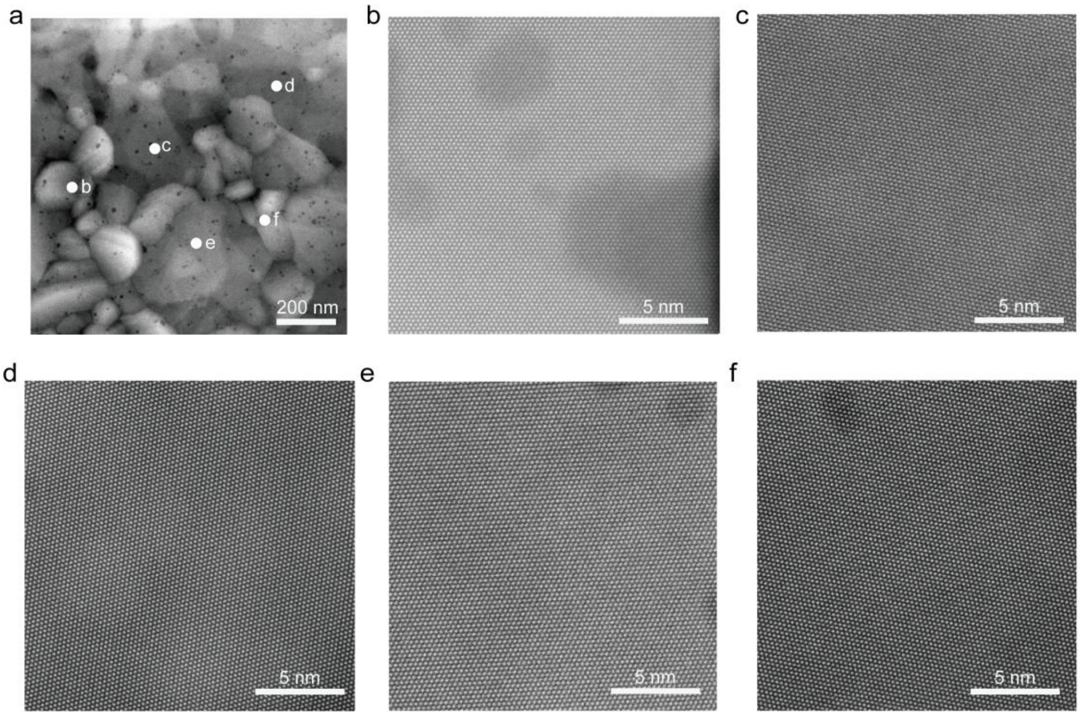

Supplementary Fig. 19 STEM characterization of $Pd_{10}Te_3$ films. a, Low-magnification STEM image of polycrystalline $Pd_{10}Te_3$. **b-f**, Atomic-resolution HAADF-STEM images of $Pd_{10}Te_3$ at different position.

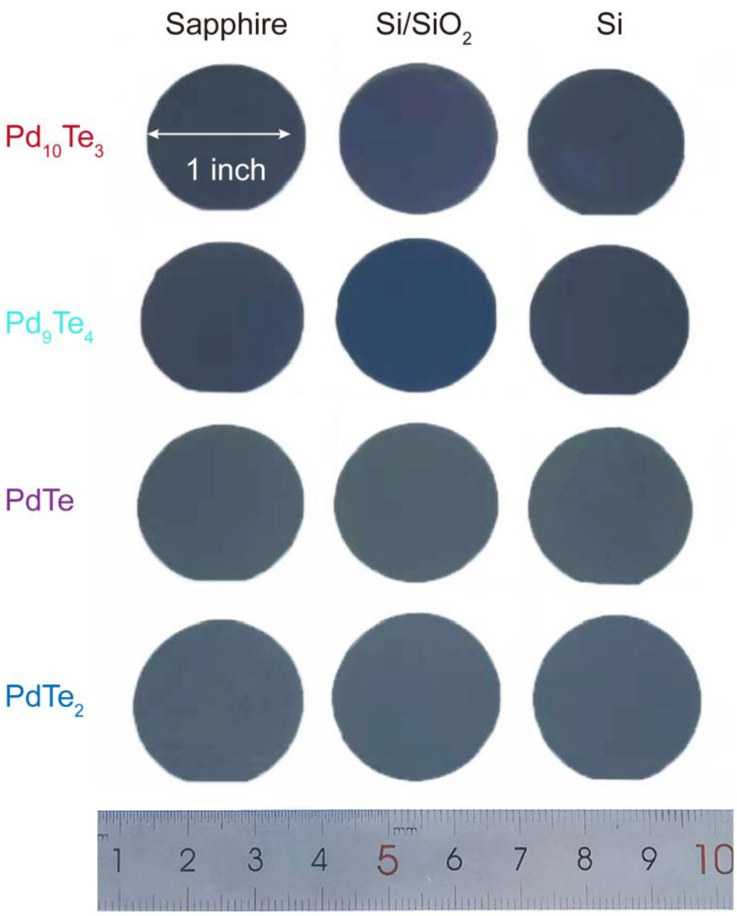

Supplementary Fig. 20 Optical images of the 2.5-cm (1-inch) $Pd_{10}Te_3$. Pd_9Te_4. PdTe and $PdTe_2$ wafers on substrates of sapphire, amorphous silicon/ silicon dioxide, and silicon.

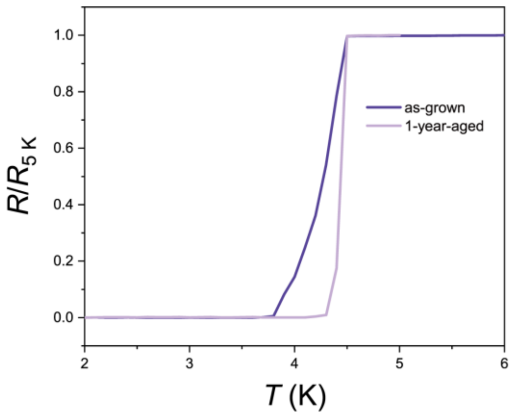

Supplementary Fig. 21 Variable temperature resistances of as-grown and 1-year-aged PdTe.

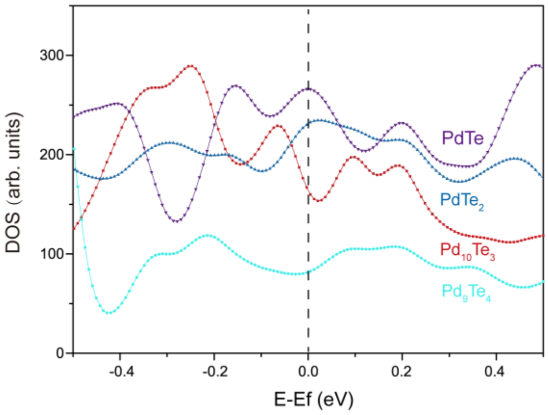

Supplementary Fig. 22 The density of states of $Pd_{10}Te_3$, Pd_9Te_4, PdTe and $PdTe_2$.

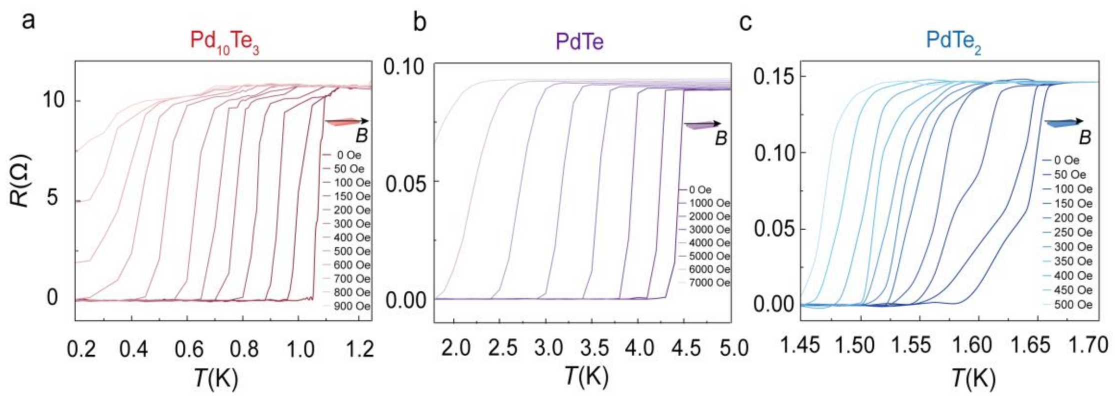

Supplementary Fig. 23 Superconductivity measurements with magnetic field applied parallel to the sample plane of a, $Pd_{10}Te_3$, **b**, PdTe and **c**, $PdTe_2$. Due to the extremely low superconducting transition temperature of Pd_9Te_4, we did not obtain the R-T curve of the Pd_9Te_4 phase under different in-plane field.

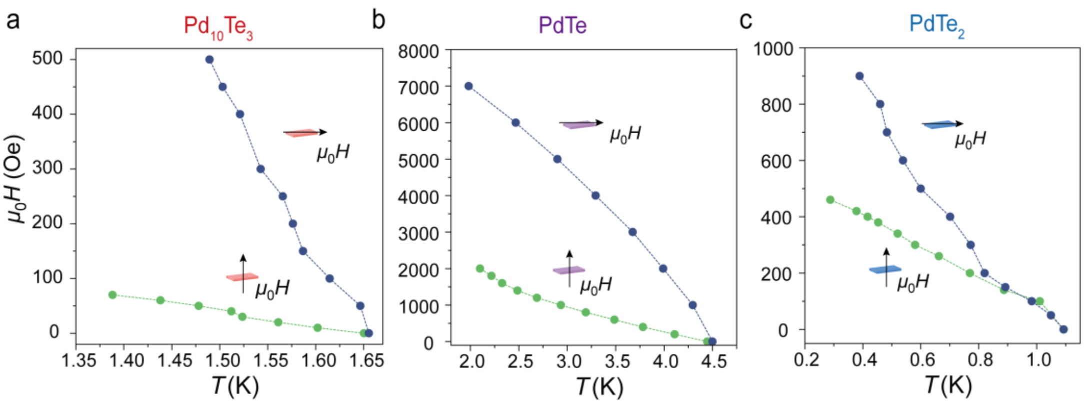

Supplementary Fig. 24 Temperature dependence of critical magnetic fields perpendicular (green circles) and parallel (blue circles) to the sample of a, $Pd_{10}Te_3$, **b**, PdTe and **c**, $PdTe_2$. The critical field in an in-plane field was enhanced compared with that in perpendicular field, indicating the 2D nature of the superconductivity of three phases.

Supplementary References

1. Anubhav Jain, Shyue Ping Ong, Geoffroy Hautier, Wei Chen, William Davidson Richards, Stephen Dacek, Shreyas Cholia, Dan Gunter, David Skinner, Gerbrand Ceder, and Kristin A. Persson. Commentary: The Materials Project: A materials genome approach to accelerating materials innovation. *APL Mater.* 1: 011002, 2013.

2.V.S. Khar'kin, R.M. Imamov, and S.A. Semiletov. The palladium telluride $Pd_{4-x}Te$ crystal structure. *Kristallografiya*, 14 :907–909, 1969.

3.W.S. Kim, G.Y. Chao, and L.J. Cabri. Phase relations in the Pd-Te system. *Journal of the Less-Common Metals*, 162:61–74, 1990.

4. P. Matkovic and K. Schubert. Kristallstruktur von Pd_9Te_4. *Journal of the Less-Common Metals*, 58:39–46, 1978.

5. L.J. Cabri, J.F. Rowland, J.H.G. Laflamme, and J.M. Stewart. Keithconnite, telluropalladinite and other palladium-platinum tellurides from the stillwater complex, montana. *Canadian Mineralogist*, 17:589–594, 1979.

6. A. Kjekshus and W.B. Pearson. Constitution and magnetic and electrical properties of palladium tellurides (PdTe - $PdTe_2$). *Canadian Journal of Physics*, 43:438–449, 1965.

7. L. Thomassen. Ueber kristallstrukturen einiger binaerer verbindungen der platinmetalle. *Zeitschrift fuer Physikalische Chemie, Abteilung B: Chemie der Elementarprozesse, Aufbau der Materie*, 2:349–379, 1929.

8. F. Gronvold and E. Rost. On the sulfides, selenides and tellurides of palladium. *Acta Chemica Scandinavica (1-27,1973-42,1988)*, 10:1620–1634, 1956.

9. W.O.J. Groeneveld Meijer. Synthesis, structures, and properties of platinum metal tellurides. *American Mineralogist*, 40:646–657, 1955.

10. S. Furuseth, K. Selte, and A. Kjekshus. Redetermined crystal structures of $NiTe_2$, $PdTe_2$, PtS_2, $PtSe_2$, and $PtTe_2$. *Acta Chemica Scandinavica (1-27,1973-42,1988)*, 19:257–258, 1965.

11. E. McCarron, R. Korenstein, and A. Wold. High pressure phase transformation studies of the system $Pd_{1-x}Rh_xTe_2$. *Materials Research Bulletin*, 11:1457–1462, 1976.

12. A. Kjekshus and F. Gronvold. High temperature X-ray study of the thermal expansion of PtS_2, $PtSe_2$, and $PdTe_2$. *Acta Chemica Scandinavica (1-27,1973-42,1988)*, 13:1767–1774, 1959.

13. M.A. Pell, Yu.V. Mironov, and J.A. Ibers. $PdTe_2$. *Acta Crystallographica, Section C: Crystal Structure Communications*, 52:1331–1332, 1996.
