# Supplementary material for: Stoichiometry-engineered phase transition in a two-dimensional binary compound
Source: Nat Commun. 2025 May 5;16:4162. doi: 10.1038/s41467-025-59429-3 (PMC12052965; doi:10.1038/s41467-025-59429-3)
Supplement: Supplementary file 2 — Description of Additional Supplementary Files [file 41467_2025_59429_MOESM2_ESM.pdf]

Description of Additional Supplementary Files

File name: Supplementary Movie 1
Description: Real-time phase evolution of 10 nm-Pd film under Te vapor exposure.

File name: Supplementary Movie 2
Description: Real-time phase evolution of 5 nm-Pd film under Te vapor exposure.

File name: Supplementary Movie 3
Description: In situ observation of spatially homogeneous nucleation and stoichiometry-controlled growth.

File name: Supplementary Movie 4
Description: Narrow nucleation window in the Pd-Te system leading to three-phase coexistence.
